# Enhancing Shear Performance of Concrete Beams Using Layered Rubberized and Steel Fiber-Reinforced Composites

**DOI:** 10.3390/ma18225076

**Published:** 2025-11-07

**Authors:** Abdulaziz S. Alsaif, Abdulrahman S. Albidah

**Affiliations:** Civil Engineering Department, College of Engineering, King Saud University, P.O. Box 800, Riyadh 12372, Saudi Arabia

**Keywords:** layered concrete beams, functionally graded beams, steel fiber reinforced concrete, rubberized concrete, shear resistance, sustainability

## Abstract

Recycling rubber and steel fibers from end-of-life tires for use in structural concrete presents a sustainable pathway to improve resource efficiency and reduce environmental impact. This study assesses the shear performance of reinforced concrete beams in which shredded tire rubber substitutes 20 vol.% of both fine and coarse natural aggregates. The effect of including recycled tire steel fibers (RSF) and industrial steel fibers (ISF), each at a dosage of 20 kg/m^3^, is also examined. The experimental program involved testing twenty-four cylindrical specimens and seven reinforced concrete beams to evaluate the mechanical and structural behavior of the proposed mixtures. A novel layered concrete configuration is also evaluated, in which rubberized (RU) concrete or steel fiber reinforced rubberized (RUSF) concrete is placed in the tensile zone, and plain (P) concrete is placed in the compressive zone. The results indicate that rubber incorporation alone reduces shear strength by 30.9% compared to P concrete. However, the inclusion of steel fibers not only compensates for this reduction but significantly improves strength and ductility. Beams fully cast with RUSF concrete exhibit a 31.9% increase in shear strength compared to P concrete. In contrast, layered beams with RUSF concrete in the bottom and P concrete in the top show a comparable performance. These findings highlight the potential of integrating steel fiber reinforced rubberized concrete and functional layering to enable the use of substantial quantities of recycled tire materials without compromising structural performance, offering a promising solution for eco-efficient construction.

## 1. Introduction

Concrete, as one of the most extensively utilized materials worldwide, consumes substantial amounts of natural resources annually. The gradual depletion of natural resources due to concrete consumption is becoming increasingly concerning, especially given the dwindling availability of natural aggregates at affordable prices [1]. Typically, natural fine aggregates (FAgg) and natural coarse aggregates (CAgg) constitute approximately 60–70% of the concrete volume, depending on the mix proportions [2]. Given that the annual concrete consumption exceeds 30 billion tons globally, even a slight replacement of natural aggregates by recycled materials would lead to significant conservation of natural resources [3]. Thus, researchers have persistently explored the integration of various recycled materials into concrete, achieving varying levels of success over the years. On the other hand, the rapid growth of urbanization and the rising need for diverse transportation modes have caused a substantial increase in the accumulation of discarded tires. The European Tyre Recycling Association has reported that roughly 300 million tires are discarded annually in the 28 EU member countries and Norway [4]. In 2023 alone, Saudi Arabia discarded approximately 20 million tires [5]. Most of these tires have accumulated awaiting re-use and/or landfill disposal, despite their high-performance constituent materials. Poor management of these tires can cause serious problems, including the risk of accidental fires and related emissions due to their high flammability [6]. Moreover, they occupy excessive landfill areas and promote mosquito breeding, which can lead to the spread of infectious diseases [7]. Hence, there is an insistent need for innovative and sustainable strategies to efficiently manage the stream of these discarded tires.

Integrating discarded tire rubber (DTR) particles into concrete offers environmental and sustainability advantages by reducing the natural resource demands of concrete consumption and by addressing the waste management issues related to the discarded tires. Over the past twenty years, significant research efforts have focused on analyzing the material characteristics of concrete that incorporates DTR particles. Known as rubberized (RU) concrete, this variant partially or entirely substitutes natural FAgg and/or CAgg with DTR particles in the form of fine rubber (FRub) and/or coarse rubber (CRub) particles. Predominantly, investigations into RU concrete, as referenced in studies [7,8,9,10,11,12,13,14,15,16,17], have utilized small-scale test models, cubes, cylinders, and prisms, to assess the composite’s material properties. The incorporation of DTR particles into concrete composites has consistently been associated with notable performance enhancements. These include increasing strain capacity, elevated flexural toughness and ductility [2,18], superior damping and energy absorption capabilities [19,20,21], greater resistance to impact loading [8], as well as a measurable decrease in the composite’s unit weight [22,23]. Nevertheless, studies have consistently indicated that incorporating DTR particles into concrete adversely affects its strength, stiffness, and workability [8,13,23]. For example, Xu et al. [24] investigated the response of RU concrete prisms under asymmetric four-point shear loading by substituting substantial proportions of FAgg and CAgg with fine and coarse rubber particles (FRub and CRub). Their findings indicated a pronounced decline in shear capacity as the rubber dosage increased; shear strength dropped by approximately 40% at a 20% replacement level, 60% at 40%, and nearly 70% at 60%, in comparison with the conventional, non-rubberized control specimens. This trend suggests a negative correlation between rubber content and shear strength, and between rubber content and stiffness [25]. These declines in performance are mainly related to the size and amount of DTR particles, with a more noticeable effect noted when CRub particles replace CAgg rather than fine particles [12,17]. The observed declines in both strength and stiffness are chiefly ascribed to the inherently weak interfacial adhesion between rubber particles and the surrounding cementitious matrix, the elevated Poisson’s ratio characteristic of DTR, and the pronounced mismatch in elastic modulus between the rubber inclusions and the remaining concrete constituents.

The incorporation of discontinuous steel fibers as an internal reinforcement mechanism in RU concrete has been explored to counteract its inherent flexural weakness. In a notable study, Ganesan et al. [26] reported that introducing 0.75% volumetric content of industrial steel fibers (ISF) into a mix where 15% of the FAgg was substituted with FRub yielded a 35% improvement in flexural strength compared with the unreinforced rubberized counterpart. Nguyen et al. [27] reported that combining DTR with ISF produced a beneficial synergistic effect on cement-based mortars: DTR improved strain capacity, while ISF enhanced post-peak behavior. Alsaif et al. [8] reported that the synergistic incorporation of recycled tire-derived steel fibers (RSF) and ISF markedly improved the toughness index of rubberized steel fiber-reinforced (RUSF) concrete cylinders. This hybrid reinforcement strategy also led to substantial gains in both flexural behavior and impact resistance in RUSF concrete prisms. Similarly, Pilakoutas et al. [28] observed that embedding RSF within the concrete matrix enhanced both strength and deformation capacity, with the mechanical reinforcement offered by RSF closely paralleling that of traditional ISF. Additionally, Alsaif and Alshannag [29] highlighted the significant cost difference, with ISF at US$2900 per ton being locally more than eight times that of RSF at US$347 per ton. Consequently, considering their comparable performance and the difference in cost, it seems that replacing ISF with RSF, either partially or fully, is a more sustainable and economically better alternative. In addition, this substitution reduces the carbon emissions associated with the manufacturing of ISF.

Despite extensive investigations into the mechanical performance of laboratory-scale RU concrete specimens, research addressing large-scale structural members, such as beams, columns, slabs, and joints reinforced with deformed steel bars and cast using RU concrete, remains comparatively limited. In one notable contribution, Ganesan et al. [26] evaluated beam-column joints fabricated with RU concrete and observed marked enhancements in crack pattern development, energy dissipation capacity, and overall ductility. Hassanli et al. [30] applied cyclic loading on RU concrete columns and identified similar enhancements. Ismail and Hassan [31] explored the flexural characteristics of self-compacting RU concrete beams, observing lower flexural strength and stiffness than for plain (P) concrete. Eisa et al. [6] conducted experiments on RU concrete beams containing FRub particles, replacing 5%, 10%, 15%, and 20% of FAgg, both with and without ISF. Their results indicated that introducing 20% FRub into the concrete mix led to a flexural strength decline of approximately 20% relative to the P concrete beams. Notably, supplementing this mixture with ISF mitigated the reduction, limiting the strength loss to roughly 9%. In a related investigation, Karthikeyan et al. [32] examined the flexural response of seven RUSF concrete beams, where sand-coated CRub particles replaced 2.5%, 5%, and 7.5% of the CAgg, and the internal reinforcement comprised either 0.5% or 1% ISF. Notably, beams incorporating 1% ISF and 7.5% CRub particles demonstrated marked improvements in several parameters: flexural strength increased by 20.8%, ductility by 40%, deformation capacity by 107.5%, and energy absorption capacity by 83.1% compared to P beams with neither FRub nor ISF.

Although numerous investigations have addressed the compressive and flexural performance of RU concrete columns and beams, research specifically focused on the shear response of RU concrete beams remains notably scarce. Ismail and Hassan [33] investigated the extent to which varying the concentrations of FRub particles, both with and without ISF, impacts the shear strength, crack resistance, and overall behavior of self-compacted RU concrete beams. They showed that increasing FRub from 0% to 25% generally reduced the beams’ shear capacity, resistance to post-diagonal cracking, and overall toughness, and enhanced deformability and lessened the beams’ self-weight. By optimizing the mixtures with FRub and ISF, they were able to alleviate the negative effects on beam strength due to the FRub, significantly enhancing ductility, toughness, and resistance to cracking. Mendis et al. [34] performed a comparative evaluation of the shear performance of RU concrete beams possessing similar compressive strengths but incorporating varying ratios of FRub particles. They discovered variations in shear capacity ranging from 10% to 15% among RU concrete beams of similar strength. They also observed that the ratio of FRub significantly reduced the ultimate shear strength of the beams. Comparable trends were observed by Al-Qurraishi et al. [35] and Ahmed et al. [36]; however, both studies demonstrated that the inclusion of ISF effectively counteracted the observed performance decline and led to notable enhancements in the shear behavior of RU concrete beams.

Recognizing that the tensile properties of concrete beams are frequently overlooked in bending design, it is practical to consider using low-strength or recycled aggregate concrete in areas subjected to tension [37]. This approach allows for natural resource conservation and waste management, as well as the possibility of reduced cement content, improved structural performance, and mass optimization. This technique, known as functionally graded or layered materials, has been employed previously in reinforced concrete beams. For instance, Lapko et al. [38] reported that a dual-layer beam with a lower layer of normal-strength concrete (25.4 MPa) and high-strength concrete (115.4 MPa) in the upper layer exhibited bending characteristics comparable to those of a beam constructed entirely from high-strength concrete. In a recent study [39], the authors of this study also investigated the flexural performance of fully cast P, RU, and RUSF concrete beams, along with the performance of layered concrete beams. The layered concrete beams were cast using two different concrete mixtures, with P concrete in the top layer and RU or RUSF concrete in the bottom layer. The authors observed that the layered concrete beam combining RUSF and P concrete outperformed the beam that was fully constructed with P concrete in flexural strength, toughness, and displacement ductility ratio by 9.9%, 12.9%, and 24.4%, respectively.

Research focusing on the shear response of layered RU concrete beams remains scarce. To date, only Ataria and Wang [37] have specifically examined this configuration, evaluating two-layer beams composed of P concrete in the upper one-third and RU concrete in the lower two-thirds. Their findings indicated that the layered system exhibited reduced shear capacity compared with a monolithic beam composed solely of plain concrete.

The foregoing review highlights that existing investigations have predominantly evaluated the influence of substituting FAgg with FRub on the shear behavior of RU concrete beams. Notably absent from the literature is any systematic assessment of hybrid reinforcement using both RSF and ISF for this application. Accordingly, further inquiry is warranted to elucidate the effects of concurrently replacing FAgg and CAgg with FRub and CRub, respectively, on the shear response of RU and RUSF concrete beams. In addition, the shear performance of layered beam configurations, comprising P concrete in the tension zone and RU or RUSF concrete in the compression region, remains largely unexplored, underscoring a significant research gap.

## 2. Research Significance and Novelty

While earlier work performed by the authors of this study [39] examined the flexural behavior of layered RU and RUSF concrete beams, the present work offers a distinct advancement by concentrating on shear performance, which is a critical factor governing the failure of structural beams that has received very limited attention in relation to layered and recycled-fiber concrete. To the best of the authors’ knowledge, this is the first comprehensive investigation into the shear behavior of RU concrete beams incorporating both RSF and ISF, thereby addressing a key gap in sustainability-driven reinforcement strategies.

The research is an attempt to complement the efforts in achieving sustainable and greener construction materials through the layered concrete concept without compromising the structural performance of the members. This practice can (i) reduce the use of natural resources, i.e., virgin aggregates, (ii) maximize the use of waste materials, i.e., discarded tires, thereby eliminating their corresponding environmental impact, and (iii) produce cost savings associated with aggregate extraction and waste landfilling. Furthermore, this study assesses layered beam configurations in which P concrete is placed in the compression zone and RU or RUSF concrete is positioned in the tensile zone under shear-critical conditions, a configuration not previously reported in the literature. The research also evaluates the combined effect of substituting both FAgg and CAgg with rubber particles on shear response, an aspect that has scarcely been systematically addressed before. By extending the layered composite concept to shear-critical applications, the present study advances current knowledge and provides practical insights for optimizing both sustainability and structural performance in reinforced concrete beams.

## 3. Experimental Campaign

### General

**Figure 1:**
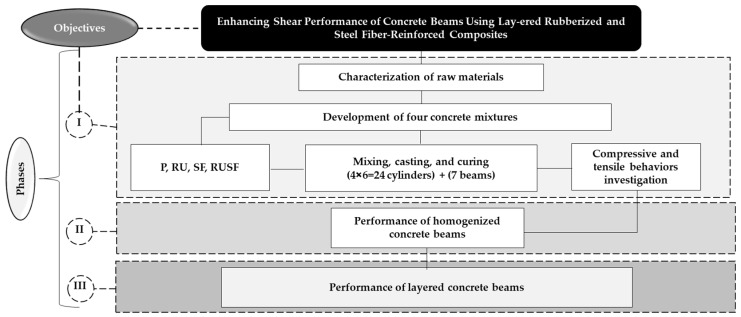
Experimental campaign design: a schematic representation.

The experimental program was structured into three sequential phases, as depicted in Figure 1. The first phase involved detailed characterization of all constituent materials, followed by the preparation of four concrete mixes: P, RU, steel fiber-reinforced (SF), and RUSF concrete. A total of twenty-four cylindrical specimens (100 mm diameter × 200 mm height) and seven beam specimens (1500 mm length × 120 mm width × 180 mm depth) were fabricated. Of these, four beams were cast using homogeneous mixtures, while the remaining three employed a layered configuration. After 28 days of wet curing, the cylinders were tested under uniaxial compression and indirect tension (three specimens per test per mixture). The second phase focused on quantifying the shear capacity of the homogeneous beam specimens. In the final phase, the shear behavior of the layered beams was examined and benchmarked against the homogeneous beam results. Detailed methodologies for each phase are provided in subsequent sections. It is important to note that, in all rubberized mixtures (RU, RUSF), both FAgg and CAgg were partially replaced at a fixed 20% level by FRub and CRub particles. The SF and RUSF mixtures additionally incorporated a total steel fiber dosage of 40 kg/m^3^, comprising equal proportions of ISF and RSF (20 kg/m^3^ each). These specific proportions were selected based on extensive prior characterization by the authors [8,13,23,39], where various levels of rubber substitution and steel fiber content were systematically investigated under compressive, flexural, and impact loading, as well as in durability and restrained shrinkage contexts. Across these studies, the combination of 20% rubber replacement and 40 kg/m^3^ hybrid steel fibers was consistently identified as the optimal compromise for achieving enhanced tensile performance, crack resistance, and acceptable workability in rubberized mixtures. Similar synergistic effects of combining ISF and RSF were also reported by Hu et al. [40], who observed significant improvements in both strength and ductility. These cumulative findings, including the authors’ own publications, provided a strong performance-based rationale for adopting the 20% rubber content and 40 kg/m^3^ hybrid fiber dosage in the present shear investigation.

## 4. Materials

### 4.1. Aggregates

**Figure 2:**
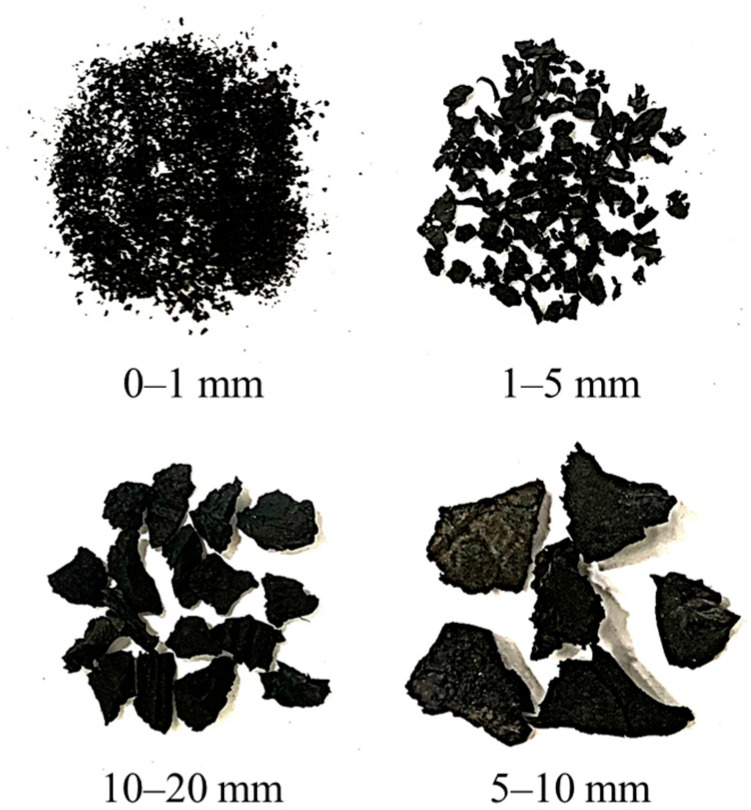
Images of rubber aggregates.

**Figure 3:**
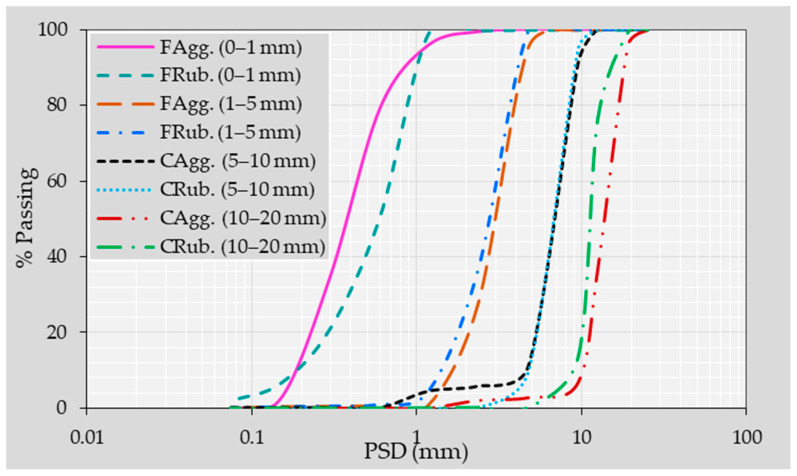
Size distribution of natural and rubber aggregates.

All concrete mixtures were produced using regionally available raw materials. The CAgg consisted of crushed limestone, graded into 5–10 mm and 10–20 mm fractions, exhibiting a bulk specific gravity (BSG) of 2.62, water absorption (WA) of 0.2%, and a loose bulk density (LBD) of 1575 kg/m^3^. The FAgg comprised well-graded sand (0–1 mm) and crushed limestone (1–5 mm). The sand possessed a BSG of 2.65, WA of 0.3%, and LBD of 1605 kg/m^3^, while the crushed limestone displayed a BSG of 2.65, WA of 2.5%, and LBD of 1650 kg/m^3^. Recycled tires were mechanically shredded by a local supplier (Ecofix Industrial & Trading Co., Ltd., Riyadh, Saudi Arabia) to produce FRub and CRub particles matching the sizes of the replaced FAgg and CAgg (see Figure 2). These were used as substitutes for FAgg and CAgg in a 1:1 volumetric ratio. Both FRub and CRub particles had a BSG of 0.82, a WA of 1.3%, and an LBD of 430 kg/m^3^. Figure 3 presents the grading curves of all aggregates.

### 4.2. Fibers

**Figure 4:**
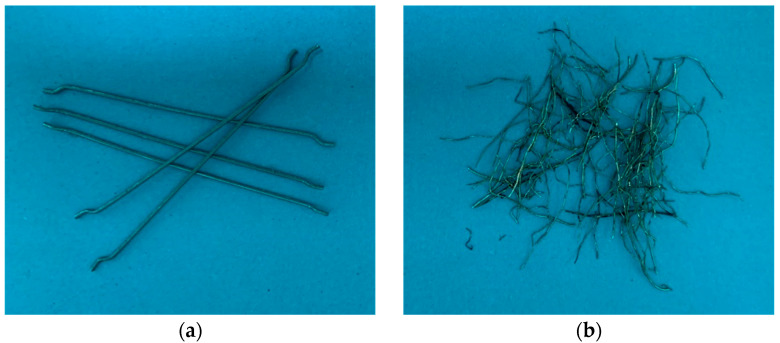
Photographs depicting (**a**) ISF and (**b**) RSF.

A blend of two categories of steel fiber was utilized in this investigation: ISF, Figure 4a, and RSF, Figure 4b. The RSF was sourced from a local company (Ecofix Industrial & Trading Co., Ltd., Riyadh, Saudi Arabia) following a multi-step recycling process. Firstly, post-consumer tires were collected and mechanically shredded into chunk-like rubber pieces using specialized machinery. These then underwent magnetic separation to extract the RSF from the rubber and other components. It is clear in Figure 4 that the ISF had consistent physico-mechanical properties characteristic of engineered steel fibers. The ISF were hooked-end and uniformly shaped with a fixed length of 60 mm, a diameter of 0.75 mm, and a tensile strength of approximately 1250 MPa. By contrast, the RSF exhibited a range of properties, with lengths ranging from 10 to 60 mm, diameters between 0.195 and 0.380 mm, and tensile strength in the range of 850–2400 MPa.

### 4.3. Longitudinal Steel Bars

The beam specimens were reinforced with four deformed steel bars: two 12 mm diameter bars positioned in the tensile zone at the bottom and two 8 mm diameter bars located in the compressive zone at the top. The mechanical characteristics of these reinforcement bars, determined through testing in accordance with ASTM A370 [41], are summarized in Table 1.

### 4.4. Details of Mixtures

As detailed in Table 2, four distinct concrete mixtures were prepared for this study (P, RU, SF, and RUSF). Mixture P was composed of Type-1 Portland cement, FAgg and CAgg, a high-range water-reducing admixture (HRWRA), and mixing water (W). Mixture RU was similar but included FRub and CRub, replacing 20 vol.% of the FAgg and CAgg. Mixtures SF and RUSF mirrored mixtures P and RU, respectively, but with the addition of a blend of 20 kg/m^3^ of ISF and 20 kg/m^3^ of RSF. It is worth highlighting that the percentage of rubber replacement and the content of steel fiber in RU and RUSF mixes were based on the results of the authors’ earlier research [8,13,23], which demonstrated improved tensile strength, flexural strength, ductility, and toughness.

In accordance with ASTM C192M [42], the concrete mixing process started by pouring the HRWRA into the W to generate a W-HRWRA solution. The CAgg and CRub were then placed in the mixer along with some of the W-HRWRA solution. The mixer was then operated briefly before the FAgg, FRub, cement, and remaining W-HRWRA solution were gradually introduced while mixing continued. After all constituents were introduced into the mixer, the batch was mixed for three minutes, paused for a two minute rest period, and subsequently remixed for an additional two minutes. For the SF and RUSF mixtures, the steel fibers were manually dispersed into the mixture during the final two minute mixing interval, followed by a further two minutes of blending to ensure uniform fiber distribution.

**Figure 5:**
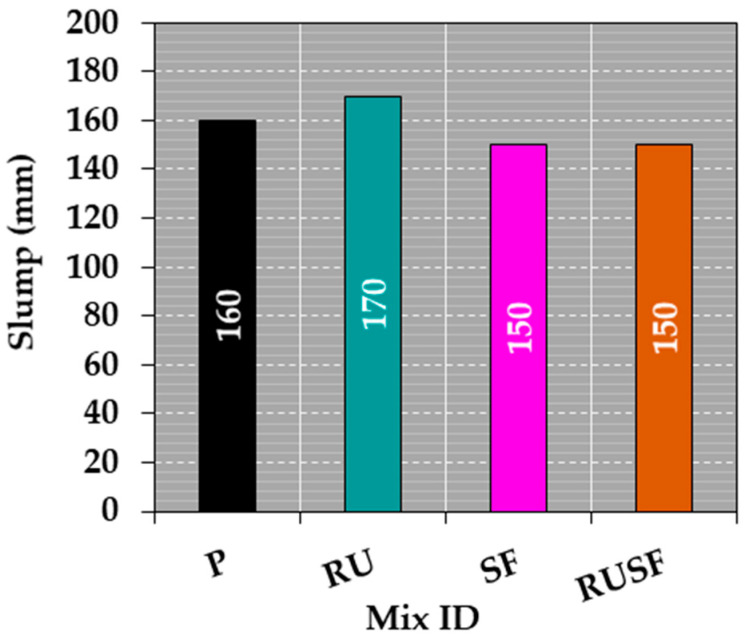
Slump values for all concrete mixtures.

It is relevant to note that the HRWRA dosage was adjusted to facilitate casting of the concrete beam mixes incorporating rubber and steel fibers. In previous studies by the authors [8,12,13,22,23], it was noted that the presence of both rubber and steel fibers increases particle friction in the fresh mix, thereby reducing its workability. To counteract this effect, the HRWRA dosage was increased accordingly. As illustrated in Figure 5, the measured slump values were 160 mm for Mix P, 170 mm for Mix RU, and 150 mm for both SF and RUSF mixes. Notably, all mixtures achieved acceptable workability despite the inclusion of rubber and steel fibers. This was achieved through adjustments to the HRWRA dosage for each mix: 2.8 L/m^3^ for P, 3.5 L/m^3^ for RU, 4.4 L/m^3^ for SF, and 5.3 L/m^3^ for RUSF, as indicated in Table 2. It is noteworthy that the casting of both homogeneous and layered concrete beams proceeded smoothly, without any issues related to segregation, handling, or finishing.

### 4.5. Details of Test Specimens

**Figure 6:**
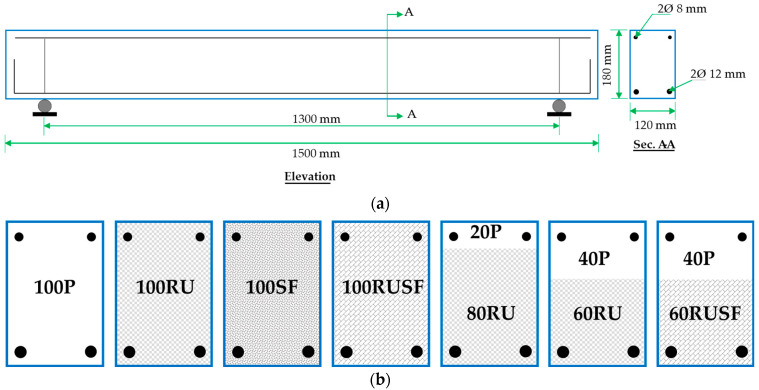
Beam details: (**a**) typical elevation; (**b**) section A-A.

To achieve the objectives of this study, seven slender reinforced concrete beams with fixed longitudinal steel reinforcement, were fabricated to examine variations in (1) the influence of the section composition used, either homogenous or layered; (2) the type of concrete mixture used to fill the beam cross-section; and (3) the thickness of each concrete layer in the case of the layered concrete method. Only one beam was cast and tested for each configuration due to the substantial material and labor demands. This approach is consistent with proof-of-concept studies, where the primary objective is to capture behavioral trends in novel structural systems. Details of the beam geometry, reinforcement, and concrete mixture types and thicknesses are shown in Table 3 and Figure 6. The number preceding the mixture ID in the beam designation column in Table 3 indicates the percentage of beam cross-sectional area containing the specified mixture. The first four beams (100P, 100RU, 100SF, 100RUSF) were cast with homogenous concrete types P, RU, SF, and RUSF, respectively, for their full depth. The remaining three beams were cast following the layered material method. The fifth beam, designated 80RU20P, was cast using a two-layer configuration in which RU concrete occupied the lower 80% of the beam’s cross-sectional depth, while the remaining upper 20% consisted of P concrete. The sixth beam, labeled 60RU40P, followed the same construction sequence but with the RU concrete reduced to 60% of the cross-section, and the P concrete increased to the upper 40%. The seventh beam, 60RUSF40P, followed the same approach as the sixth beam but used RUSF concrete for the bottom layer.

The concept of layered concrete beams was adopted to optimize structural efficiency by selectively incorporating RU or RUSF mixtures within the cross-section, in alignment with strategies reported in earlier investigations [39]. Given that the inclusion of rubber particles adversely affects the mechanical integrity of concrete, resulting in diminished flexural and shear resistance, the layered configuration was employed to mitigate these drawbacks while capitalizing on the benefits of rubber particles. Since the top layer is critical to the compressive strength (i.e., shear contribution in the compression zone), it was fabricated using high-compressive-strength type P concrete. The thickness was selected to maximize the volume of rubberized concrete in the cross-section in a way that avoided reducing the shear strength (due to the shear contribution from the compression zone or from aggregate interlock).

### 4.6. Casting and Curing Method

Each concrete mixture was initially cast into six cylindrical molds. Three cylinders of each mix were tested for compressive behavior, and three cylinders were reserved for evaluating split tensile strength. This replication per mix aligns with common standard practices in exploratory concrete materials research. For layered beams, the bottom layer was poured first and thoroughly vibrated and compacted. The top layer was then cast while the bottom layer was still fresh, mostly within 30 min of completing the bottom layer. The top layer was also effectively vibrated, and its surface was carefully finished. The casting of the two layers of concrete while both were fresh resulted in a monolithic cross-section showing no signs of interface or joints that separate the two layers of concrete. This is clearly evident from the beam photos presented later in Section 5.2.1 (Failure modes).

After the cylinders and beams were poured, they were covered with damp burlap and maintained in laboratory conditions at a temperature of 24 ± 2 °C for two days. They were then removed from the molds and rewrapped in damp burlap, which was regularly moistened, for an additional 26 days to ensure adequate curing.

### 4.7. Experimental Setup and Instrumentation

The compressive and indirect tensile responses of the cylindrical specimens were assessed following the protocols outlined in ASTM C39/C39M-21 [43] and ASTM C496/C496M-17 [44], respectively.

**Figure 7:**
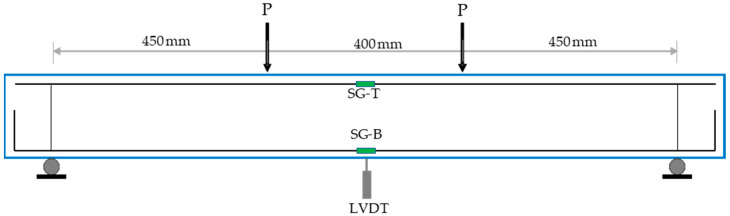
Shear test configuration.

The shear behavior of the beam specimens was subsequently examined using a four-point bending configuration. It is important to note that the beams were intentionally constructed without transverse shear reinforcement along the shear span, apart from two 8 mm diameter stirrups positioned at the supports to secure the top longitudinal reinforcement. Rigid steel cylinders were utilized both for loading points and supports. The supports were located to allow for an overhang of 100 mm at each end, producing an effective span of 1300 mm, with the middle point loads set 400 mm apart. The testing machine was operated in displacement-controlled mode, initially applying a load at 0.5 mm/min until the beam reached its peak load, then the loading rate was doubled. The reduced initial loading rate facilitated precise monitoring and documentation of crack initiation and propagation. Mid-span vertical deflections were captured using a linear variable differential transducer (LVDT) placed at the center of the beam. To record strain responses, an electrical resistance strain gauge (SG-B) was affixed to one of the bottom longitudinal reinforcement bars at mid-span to monitor tensile strain, while a second gauge (SG-T) was installed on a top bar at the same location to capture compressive strain (Figure 7). It is worth highlighting that no visible signs of interlayer slip or delamination were observed during testing of the layered beams, indicating a sufficient bond at the interface under monotonic loading conditions. This is clearly evident from the beam photos presented later in Section 5.2.1 (Failure modes), which show a monolithic cracking pattern along the beam depth without evidence of interfacial debonding. While these qualitative findings suggest that slip did not govern shear transfer in the tested configurations, it should be noted that interfacial slip was not directly measured in this study. Therefore, its potential influence cannot be fully excluded, and future studies are recommended to perform quantitative assessments of interlayer bond strength and its contribution to structural performance.

## 5. Results and Discussion

### 5.1. Mechanical Properties of Various Concrete Mixes

**Figure 8:**
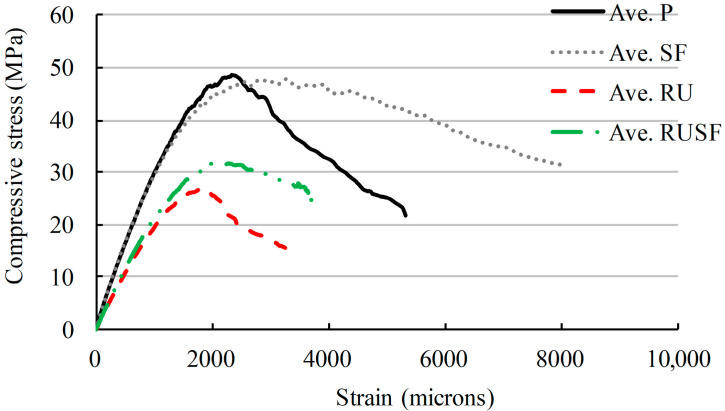
Average stress–strain relationships for the different concrete mixtures.

The compressive behavior of the four concrete mixtures (i.e., P, SF, RU, RUSF) was investigated. The average stress–strain relationships based on three samples of each concrete type are compared in Figure 8.

The 28-day compressive strength, modulus of elasticity, strain at peak strength, and splitting tensile strength are summarized in Table 4. The P and SF concretes displayed similar stress–strain behavior until the peak load was reached, with almost identical modulus of elasticity, and compressive strengths of 48.4 MPa for the P concrete and 48 MPa for the SF concrete. However, the introduction of rubber to the mix resulted in a decrease relative to the P concrete in both the modulus of elasticity and peak load. The average compressive strength of the RU concrete was 27 MPa, only 55.7% of the P concrete value. However, the addition of steel fibers to the rubberized mix recovered some of the lost strength, increasing the average compressive strength to 32.2 MPa for the RUSF mix. The largest value of strain at peak strength was achieved by the SF concrete mix, followed by the P, RUSF, and RU mixes. This highlights the positive effect of the added steel fibers on strain capacity, and the opposite trend for rubber content alone. The average splitting tensile strengths of mixes P, RU, SF, and RUSF were 5.53, 3.95, 7.0, and 5.95 MPa, respectively. These trends were generally similar to the compressive strength for mixes without fibers. However, the splitting tensile strengths of mixes incorporating steel fibers were enhanced significantly and exceeded the strength of mix P.

### 5.2. Shear Performance of the Beams

#### 5.2.1. Failure Modes and Cracking Patterns

**Figure 9:**
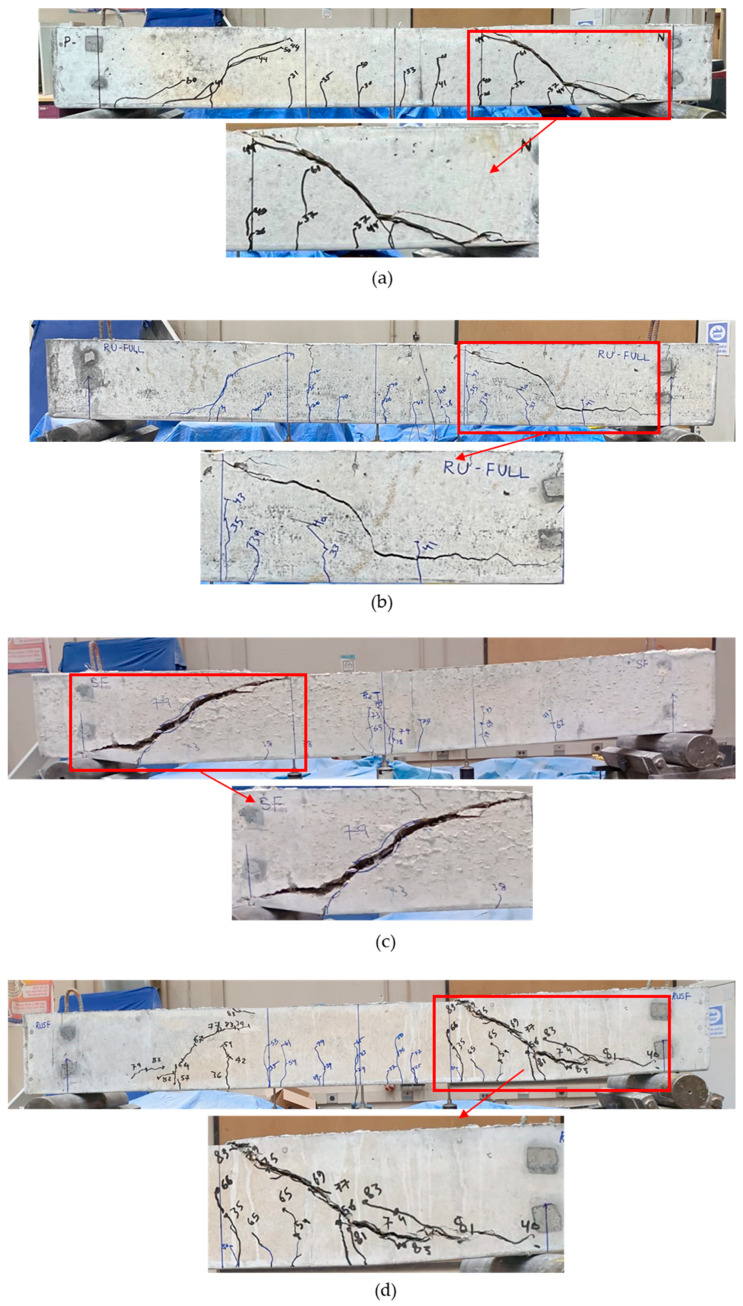
Cracking distribution for homogenous beams: (**a**) 100P; (**b**) 100RU; (**c**) 100SF; (**d**) 100RUSF.

Beams cast of homogenous material, 100P, 100RU, 100SF, and 100RUSF, exhibited a typical shear failure mode characterized by a major diagonal crack running between the loading points and beam supports (Figure 9). Observations of the cracking behavior for the 100P beam indicated that cracking was initiated by a series of small flexural cracks (Figure 9a) in the pure bending region (i.e., middle region of the beam) at loads of about 25–41 kN. Two major shear cracks were observed at both sides of the beam, initiated at about 41 kN; one of these controlled the beam behavior until failure at 63.4 kN. The cracking behavior of the 100RU beams was generally similar to that of the 100P beam, except that the first flexural crack occurred at a lower load of about 20 kN, and cracks were thinner (Figure 9b). A shear crack that failed the beam spanned from the loading point to the right-hand support at a load of 44 kN. Beam 100SF showed less cracking than beams 100P and 100RU, with flexural cracks initiating at 37 kN loading (i.e., greater than for the 100P and 100RU beams). Only one shear crack initiated at the left-hand side of the beam (Figure 9c) and controlled the beam behavior until failure at a load of 88.5 kN. It is quite clear that the steel fibers delayed crack initiation compared to beams without steel fibers. The 100RUSF beam cracking behavior (Figure 9d) was similar to that of beams 100P and 100RU, where a set of flexural cracks initiated in the mid-span zone at different loads starting at 24 kN. Two main shear cracks formed at both sides of the beam, one of which controlled the behavior of the beam until failure at a load of 83.7 kN.

**Figure 10:**
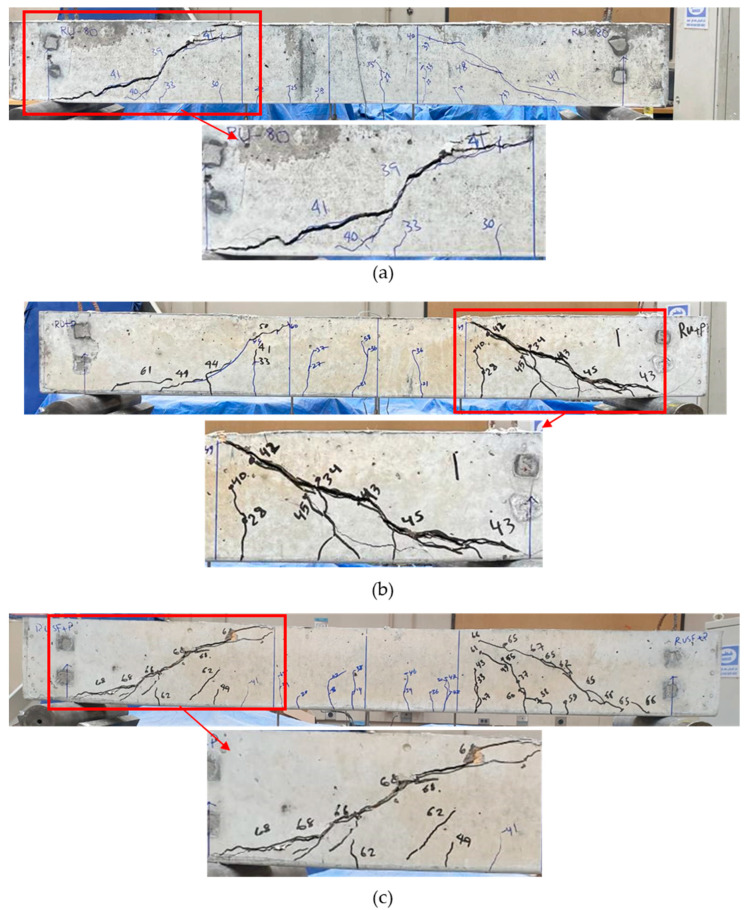
Cracking distribution for layered beams: (**a**) 80RU20P; (**b**) 60RU40P; (**c**) 60RUSF40P.

For beams 80RU20P, 60RU40P, and 60RUSF40P cast with two layers of different concrete mixes, Figure 10 shows that their modes of shear failure were all similar to those in the homogenous beams. This was to be expected, as the beams had no shear stirrups and were appropriately reinforced in flexure so that shear failure would precede flexural failure. The distribution of cracks in beam 80RU20P (bottom 80% layer of RU concrete, and top 20% of P concrete, Figure 10a) was comparable to that of the 100RU beam, with flexural cracks initiating at a similar applied load of about 22 kN. Two shear cracks initiated in the RU concrete layer at the sides of the beam at 39 kN load and continued to propagate with increasing load until the beam failed at 58.7 kN. The cracking behavior of beam 60RU40P (RU bottom layer of 60% of the beam depth, Figure 10b) was not appreciably different from that of beam 80RU20P. The initial flexural cracks were observed at 21 kN, and the first shear crack at 42 kN. The beam failed at an applied load of 63 kN. The third layered beam (60RUSF40P) with 60% RUSF concrete and 40% P concrete had cracking patterns (Figure 10c) generally similar to those of 100RUSF, with initial flexural cracks and shear cracks controlling beam behavior until failure at a load of 68.2 kN.

#### 5.2.2. Force–Displacement and Strain Development

**Figure 11:**
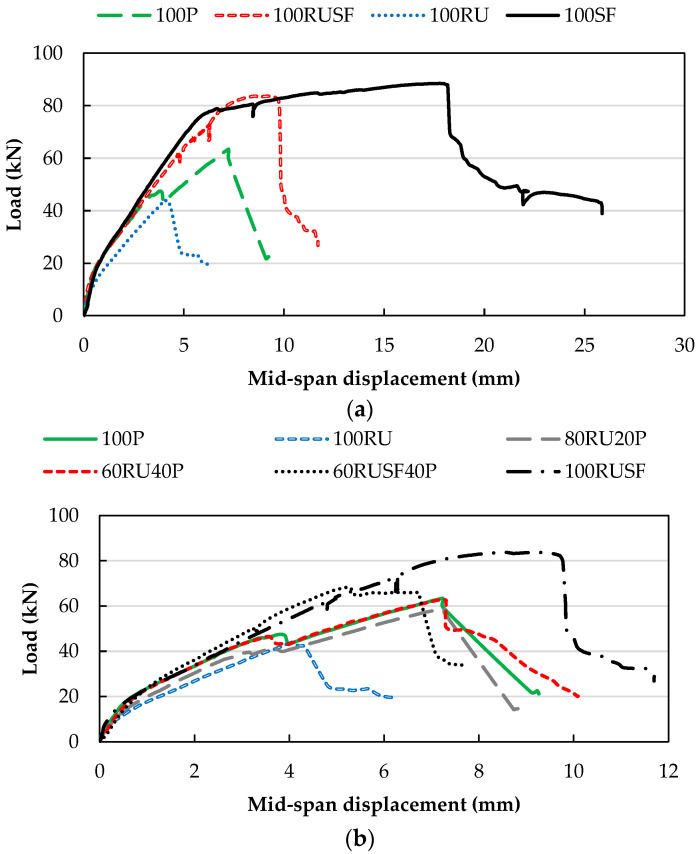
Force–displacement relationship comparison for (**a**) homogenous beams; (**b**) layered beams.

The force versus mid-span displacement relationships for the various beams are given in Figure 11. Key components of the force–deformation relationship are also summarized in Table 5, including peak load and corresponding mid-span displacement, and strain in the bottom and top longitudinal steel. Figure 11a presents the force–displacement responses of the beams cast with single-material mixtures (100P, 100RU, 100SF, and 100RUSF). At the initial loading stage, before the onset of flexural cracking, all beams exhibited comparable behavior, with the greatest stiffness observed while the section remained uncracked. However, in the post-flexural cracking phase, beams with 100RU concrete showed the lowest stiffness, followed by 100P concrete beams. The low stiffness of the 100RU concrete beam is evidently due to the low elastic modulus of rubber [36].

Beams with steel fibers, with or without rubber, had higher stiffness than 100RU and 100P beams. The fibers resist the induced principal tensile stresses developed in concrete, consistent with the higher splitting tensile strength observed for 100SF and 100RUSF concrete mixes compared to 100P and 100RU concrete mixes. Beams with steel fibers possessed greater deformation and strength capacity than their counterpart beams without steel fibers, due to their ‘stitching’ effect that allows transfer of stresses across the crack. The enhanced peak load and displacement capacity of concrete beams with steel fibers compared to their counterpart beams without fibers confirms the role of fibers in increasing the energy absorption capacity of the beams, as indicated by the larger area enclosed by the load–displacement curves in Figure 11a. The abrupt post-peak behavior is representative of typical shear failure. The strain developed in the tension steel in 100P and 100RU concrete beams was 2348 and 1936 microstrain, respectively. The observation that it did not reach the yield strain of 2732 microstrain is consistent with the observations reported in [36]. However, the higher shear strength provided by the steel fiber reinforced concrete, with or without rubber, resulted in the development of larger tensile stresses in the bottom longitudinal reinforcement bars. This was evident from the developed tensile strain exceeding the yield strain (30,241 and 5697 microstrain for beams 100SF and 100RUSF, respectively). This is also compatible with mid-span displacement for beams 100SF and 100RUSF, 17.8 and 8.6 mm, respectively. A similar observation was also noticed in tests reported in [45]. Compressive strain was very low in the 100P beam, at about 297 microstrain at peak strength. It should be noted that compressive strain values for beams 100RU, 100SF, and 100RUSF are not available due to unreliable readings.

Beams constructed with layered concrete displayed an interesting force–displacement relationship. Although the beam with 100RU concrete showed a significantly reduced flexural stiffness after cracking, beams with 20% and 40% P concrete placed in the top layer over RU concrete (i.e., 80RU20P and 60RU40P) evidenced stiffer behavior, as shown in Figure 11b. Importantly, the 60RU40P concrete beam had a pre-peak stiffness identical to the 100P concrete beam, indicating the possibility of employing RU concrete in beam construction without compromising flexural stiffness. Similarly, the 60RUSF40P concrete beam exhibited a pre-peak force–deflection curve comparable to those of both the 100P and 100RUSF beams. In other words, the addition of steel fibers to the RU concrete recovered the lost stiffness due to the rubber content. Both the homogenous and layered RUSF (i.e., 100RUSF and 60RUSF40P) had a higher strength and deformation capacity than either 100P or 100RU. Longitudinal strain at peak load for bars under tensile stress in layered beams ranged from 2014 to 2682 microstrain; the strain in bars under compressive stress did not exceed 645 microstrain.

### 5.3. Influence of Material Type on the Shear Behavior for Homogenous Beams

**Figure 12:**
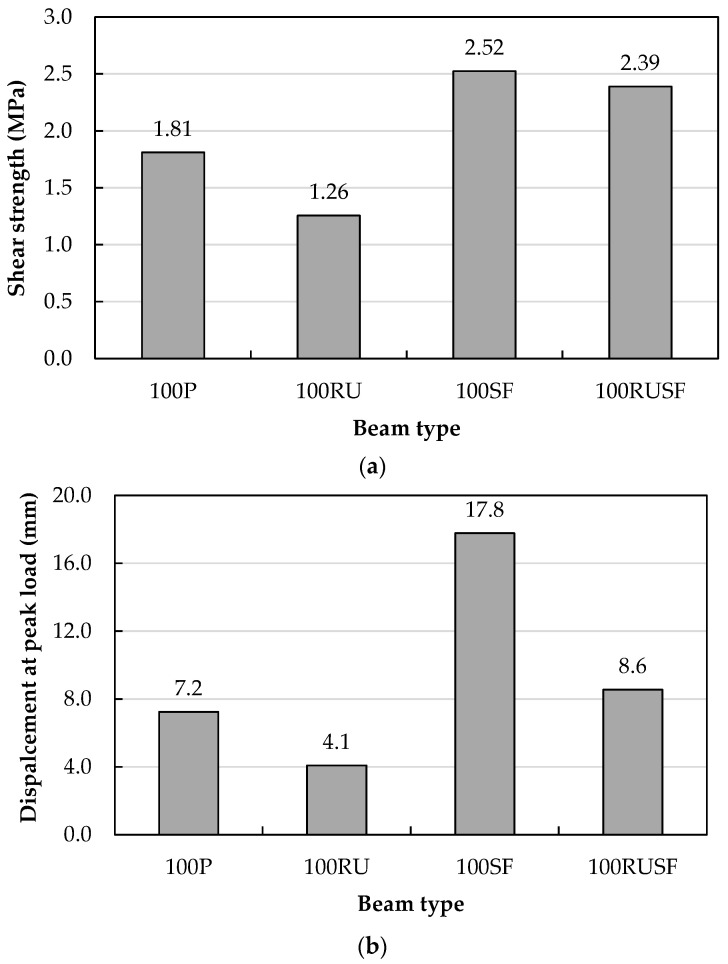
Comparisons of homogenous beams in terms of (**a**) shear strength and (**b**) mid-span displacement at peak load.

Figure 12 presents a comparison of shear strength, calculated as the peak applied load divided by the effective cross-sectional area (P/bd), and the corresponding mid-span displacement at peak load for beams fabricated using uniform material compositions throughout their depth. As illustrated in Figure 12a, introducing rubber into the concrete matrix (100RU) resulted in a 30.6% decrease in shear capacity relative to the plain concrete counterpart (100P) containing no rubber. This outcome is consistent with results in [33] in which the shear strength was reduced by about 30.5% when 25% crumb rubber was utilized in self-consolidating concrete beams. The drop in shear strength as a consequence of rubber addition to the mixture occurred for many reasons, including (i) the reduction in the compressive strength caused by the partial replacement of FAgg and CAgg by FRub and CRub, which led to a reduction in the shear strength resistance in the compression zone of the beams; (ii) the softness of the rubber may reduce frictional forces along the inclined shear cracks, and also may negatively affect the aggregate interlock; and (iii) the reduced tensile strength of RU concrete allows for the initiation of shear cracks at a lower load than in beams of P concrete without rubber, leading to the development of failure shear cracks. A study in which the substitution of natural aggregate by 40% crumb rubber caused a shear strength reduction of 29.5% [36] reached similar conclusions: the lower strength was related to the low stiffness and strength of rubber as well as the weak bonding between the concrete paste and rubber particles, facilitating crack growth and hence failure at a lower ultimate load. However, beam 100SF incorporating steel fibers raised the shear strength to 39.4% higher than the value for the 100P. Although the compressive strengths of the P concrete and SF concrete were generally similar, the splitting tensile strength for SF concrete was higher. The better tensile strength is due to the steel fibers delaying the appearance of shear cracks as they resist the principal tensile stresses developed in the beam. Following initiation of the shear crack, steel fibers also reduce the crack opening by their ‘stitching’ action, transferring tensile stresses along the crack [33]. Moreover, fibers in the concrete mixture may function as elongated aggregate particles that develop an aggregate-mortar interlock and increase frictional forces [33,46]. Interestingly, the shear strength of beams incorporating rubber with steel fibers (100RUSF) exceeded the strength of 100P beams by 31.9% and was very similar to the 100SF beam with steel fibers alone (i.e., 2.39 MPa vs. 2.52 MPa). This suggests that the presence of steel fibers not only counteracted the lowered shear strength in the rubberized concrete, but it also increased the shear strength above that of the 100P concrete.

The trend of mid-span displacement at peak strength for beams cast with a single material generally paralleled shear strength (Figure 12b). The displacement for the 100RU beam was 43.1% lower than the 100P value, but 147.2% higher for 100SF and 19.4% higher for 100RUSF beams. The relatively low shear strength of beams 100P and 100RU did not allow plastic deformation, as was evident from their tensile strains that were below the yield point. However, the large shear strengths of the 100SF and 100RUSF beams led to the development of a larger plastic strain in the tension steel, which was reflected in the observed higher mid-span displacement.

### 5.4. Influence of Layer Thickness on the Shear Behavior of Rubberized Beams

**Figure 13:**
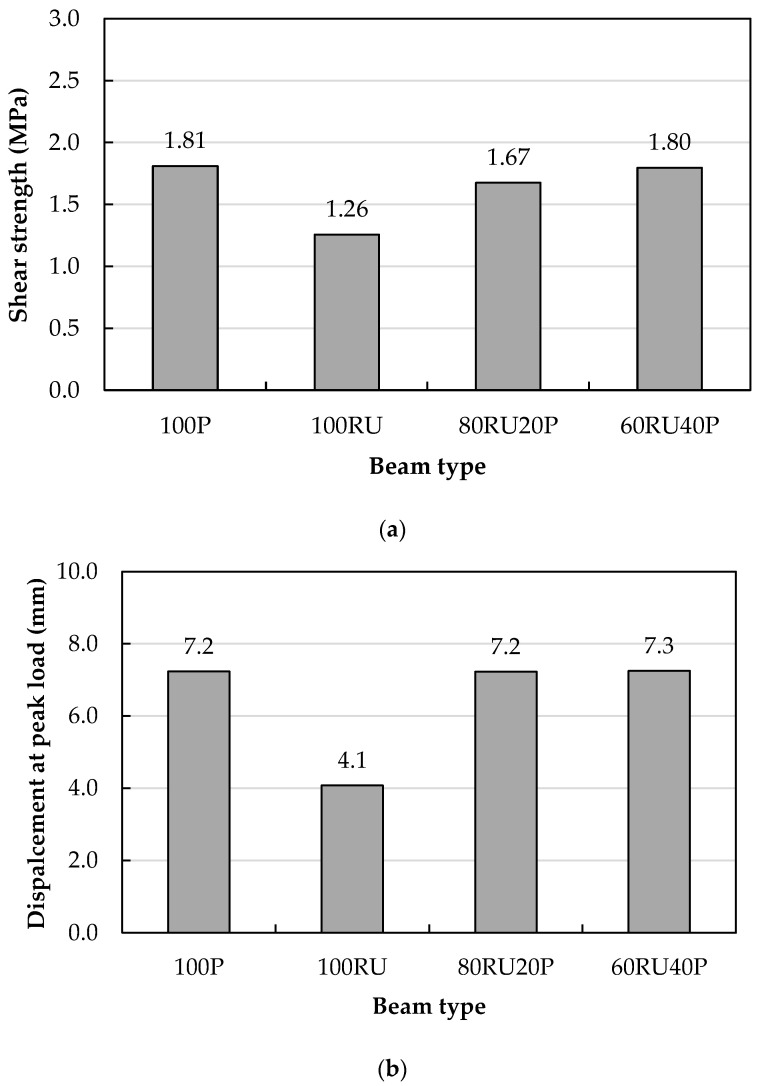
Comparison of layered beams comprising plain and rubberized concrete: (**a**) shear strength and (**b**) mid-span displacement at peak load.

Shear strength and mid-span displacement for beams with P concrete and RU concrete in layers of various thicknesses are compared with the control beams (homogenous P or RU concrete) in Figure 13. The shear strength of the 80RU20P concrete beam (top 20% depth P concrete) exceeded the 100RU beam value by 32.5% but was lower than the 100P beam (100P) by 7.7% (Figure 13a). Increasing the depth of the top layer of P concrete to 40% of the total depth (60RU40P beam) increased the shear strength to nearly the same level as 100P.

The shear strength improvement upon replacement of the RU concrete in the top part of the beam is closely linked with the shear transfer mechanism in beams. In beams without shear reinforcement, shear resistance is sourced from the concrete compression zone, aggregate interlock, and dowel action of longitudinal steel. According to [47], the shear transferred by compression zone, aggregate interlock, and dowel action are 20–40%, 33–50%, and 15–25%, respectively. The confinement and integrity of the compression zone is a key parameter that has been addressed by many researchers [48] for modeling its behavior. Therefore, it is quite clear that the replacement of the relatively weak RU concrete in the top part of the cross-section by higher compressive strength P concrete contributes to the increase in shear strength brought by the compression zone, and by aggregate interlock when the soft rubber particles are replaced by natural stiff aggregate. Comparing the results of the 100P, 80RU20P, and 60RU20P shows the sensitivity of the top layer thickness to the shear transfer mechanisms highlighted above, where increasing the top layer thickness from 20 to 40% eliminated the loss in the shear strength (due to the presence of rubber) from 7.7% to 0.55%. This outcome is aligned with the beam result obtained by Ataria and Wang [37], in which the top 33.3% of the beam is P concrete, while the remaining bottom is rubberized concrete with recycled aggregate. The lost shear strength is about 13.5% compared to the control beam. Although the present research and Ataria and Wang [37] both achieved a limited reduction in the shear strength when the layered beam concept was used, the rate of reduction in the shear strength was higher for Ataria and Wang [37]. This may be explained by the fact that they used rubber with recycled aggregate in the rubberized concrete mix, while the present research utilized rubber with virgin aggregate. The utilization of recycled aggregate can contribute to further reduction in the aggregate interlock. Generally, the observed behavior highlights the benefits of using more than one layer of material to cast the beam: the merits of RU concrete are utilized without compromising the shear or flexural performance of the beams.

The beams with layered concrete 80RU20P and 60RU40P both showed a better mid-span displacement at peak strength than 100RU, as shown in Figure 13b. Importantly, the mid-span displacements for both were similar to those of non-layered beam 100P, for the reason that casting the beams in layers enhanced the shear strength of the plain/rubberized beams to almost the same level of strength as the plain beam, while the longitudinal flexural strains were still in the elastic range (i.e., did not reach the yield point of the steel).

### 5.5. Influence of Layer Thickness on the Shear Behavior of Steel Fiber Reinforced Rubberized Beams

**Figure 14:**
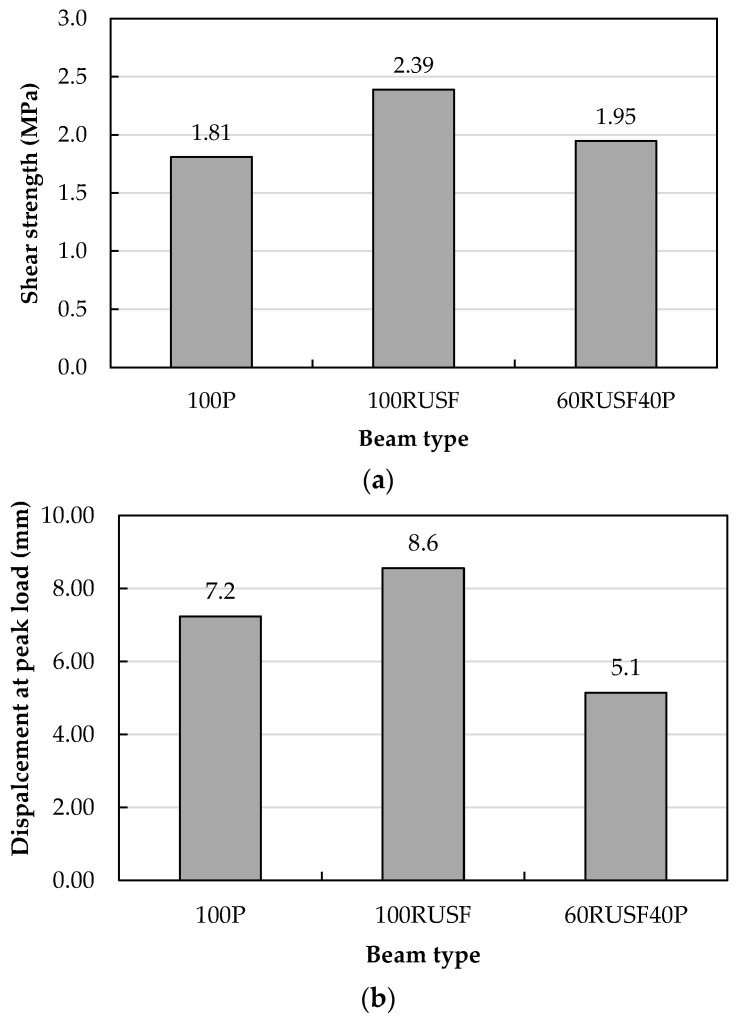
Comparisons between layered beams of plain and rubberized steel fiber reinforced concrete: (**a**) shear strength and (**b**) mid-span displacement at peak load.

In Figure 14, the shear performance of beams layered with P concrete and RUSF (60RUSF40P) is compared with that of beams fully cast with single material, 100P and 100RUSF. Replacing the top layer of RUSF concrete with P concrete caused a drop in the shear strength of the 60RUSF40P concrete beam from 2.39 to 1.95 MPa, which was still greater than the value for 100P (i.e., 1.81 MPa, Figure 14a). Referring to the shear-resisting mechanisms, it could be concluded that the compression zone did not influence the drop in shear strength of beam 60RUSF40P, since RUSF concrete and P concrete possess a similar compressive strength. Therefore, the reduction could be primarily attributable to the loss of steel-fiber bridging action in the tensile zone of the upper layer, i.e., the absence of fibers across the shear crack path in the tensile zone of the 40% P concrete layer. It is important to note the role of fibers in crack bridging and load transfer across the crack compared to concrete mixes containing no fiber. A recent study by Shafaie et al. [49], utilizing slant shear tests confirmed the beneficial effect of fibers in improving the shear bond strength. Figure 14b shows a lower displacement for the layered beam than for beams 100P and 100RUSF fully cast with a single material. However, the peak strength of the beam 60RUSF40P was followed by a small strength decrease and continued to carry the load up to a displacement comparable to the displacement at peak strength reached by beam 100P.

### 5.6. Prediction of the Shear Strength for Beams of Homogeneous Material

The shear strength for the beams without fibers, 100P and 100RU, was predicted and compared against the experimental results. Three models were used to predict the shear strength: ACI 318 [50], Eurocode [51], and Zsutty [52] as given by Equations (1)–(3), respectively.

(1) $$V_{c}=\left(0.16\sqrt{f_{c}^{\prime }}+17\rho_{w}\frac{V_{u}d}{M_{u}}\right)b_{w}d\leq 0.29\sqrt{f_{c}^{\prime }}b_{w}d$$

(2) $$V_{R,c}=\left[0.18k{\left(100\rho_{1}f_{ck}\right)}^{\frac{1}{3}}+0.15\sigma_{cp}\right]b_{w}d\;;\;k=1+\sqrt{\frac{200}{d}}\leq 2$$

(3) $$V_{c}=2.17{\left(f_{c}^{\prime }\rho_{t}\frac{d}{a}\right)}^{1/3}\;b_{w}d\left(a/d\geq 2.5\right)$$

where $f_{c}^{\prime }$ ($f_{ck}$) is the concrete compressive strength; $\rho_{w}(\rho_{1}or\rho_{t})$ is the tension flexural reinforcement ratio; $b_{w}$ is the beam width; *d* is the effective depth of the beam; *M_u_* is the ultimate moment; *V_u_* is the ultimate shear force; $\sigma_{cp}$ is the axial compressive stress; and *a* is the shear span length. The predicted results are summarized in Table 6.

It is seen from Table 6 that the predicted to experimental shear ratio for the beam 100P was 0.65, 0.79, and 0.70 for the ACI 318 [50], Eurocode [51], and Zsutty [52] models, respectively. Similarly, for the rubberized beam 100RU, the shear strength was also underpredicted with a predicted to experimental shear ratio of 0.72, 0.94, and 0.84, for the ACI 318 [50], Eurocode [51], and Zsutty [52] models, respectively. It is evident that the Eurocode prediction was the best among other models. The underprediction is within expectation given that codes are generally conservative for safety considerations.

For the steel fiber reinforced concrete beams 100SF and 100RUSF, the empirical equation of Narayanan and Darwish [53] was utilized to predict their shear strength as given in Equation (4) through Equation (6):

(4) $$V_{u}=\left[e\left[0.24f_{spfc}+80\rho \frac{d}{a}\right]+v_{b}\right]b_{w}d$$

(5) $$v_{b}=0.41\tau F$$

(6) $$f_{spfc}=\frac{f_{cuf}}{20-\sqrt{F}}+0.7+\sqrt{F}$$

(7) $$\;F=\frac{L_{f}}{D_{f}}V_{f}d_{f}$$

where e is arch action factor taken as 1 for a/d > 2.8; $f_{spfc}$ is the calculated splitting tensile strength for fiber reinforced concrete; τ is the average fiber matrix interfacial bond stress taken as 4.15 MPa following recommendations of Swamy et al. [54]; $f_{cuf}$ is the cube compressive strength of concrete (assumed as 1.25 $f_{c}^{\prime })$; $L_{f}$ is the fiber length; $D_{f}$ is the fiber diameter; and $V_{f}$ is the volume fraction of fibers; $d_{f}$ is the bond factor (0.5, 0.75, and 1 for round, crimped, and indented fibers, respectively). In the present prediction, the fibers’ factor F was calculated as a combination of the two types of fibers considered, ISF and RSF, each based on its properties. However, due to the variability of the diameter and length of RSF, the average values were utilized. The bond factor $d_{f}$ was selected as 0.5 for RSF considering its round shape, and 1 for the ISF considering its hooked-end shape, as adopted in previous research of Imam et al. [55].

It is important to note that the shear strength prediction using Narayanan and Darwish [53] was made twice for each beam, depending on the way of considering the splitting tensile strength $f_{spfc}$: (i) utilizing the estimated value from Equation (6) and (ii) utilizing the experimental value. The shear strength prediction along with the comparison with the experimental results are summarized in Table 7. It is seen that the first prediction of the model (based on the estimated splitting tensile strength) predicted the experimental results with relatively good accuracy, with a predicted to experimental shear strength ratio of 0.79 and 0.74 for the beams 100SF and 100RUSF, respectively. However, the prediction accuracy was significantly enhanced when the experimental splitting tensile strength was employed in the Narayanan and Darwish [53] equation as a replacement the for value given by Equation (6). The predicted strength was almost the same as the experimental strength, with a predicted to experimental shear strength ratio of 1.04 and 1.00 for the beams 100SF and 100RUSF, respectively. The superior performance of the second prediction over the first prediction can be associated with doubt about the estimated average value of the fiber factor for the recycled fibers, since the fibers were highly variable in terms of length and diameter, and the selection of the average values may not be a proper assumption.

## 6. Conclusions, Limitations, and Future Work

The present investigation studied the shear performance of beams incorporating fine and coarse rubber particles replacing natural aggregate with and without steel fibers. The concept of layered concrete (i.e., functionally graded materials) was utilized to maximize the use of waste tire rubber without compromising the shear performance of the beams. The outcomes of this study included:

- Replacing 20% of fine and coarse aggregates with waste tire rubber reduced the cylindrical compressive strength by 44.2% and beam shear strength by 30.9% compared to plain concrete, highlighting the structural limitations of rubberized concrete.
- Incorporating 20 kg/m^3^ of ISF alongside 20 kg/m^3^ of RSF into rubberized concrete mixes improved mechanical performance, increasing compressive strength by 19.3%, tensile strength by 50.6%, and shear strength by 90.2% compared to rubberized concrete without fibers.
- Both the homogeneous and layered RUSF beams (i.e., 100RUSF and 60RUSF40P) exhibited superior shear strength and deformation capacity compared with both 100P and 100RU. In particular, the fully rubberized steel fiber-reinforced beam (100RUSF) outperformed the plain concrete beam (100P), demonstrating increases of 31.9% in shear strength and 19.4% in displacement at peak load.
- Substituting the upper 20% of the cross-section in a fully rubberized beam with plain concrete increased the shear strength from 1.26 MPa (100RU) to 1.67 MPa (80RU20P), reducing the strength deficit to only 7.7% relative to the plain concrete beam (100P). Further increasing the plain concrete layer to 40% (60RU40P) fully restored the shear capacity to a level comparable with 100P.
- Among the analytical models evaluated, the Narayanan and Darwish shear prediction model demonstrated excellent accuracy when the experimentally measured tensile strength was used as input. This highlights its practical suitability for reliably estimating the shear capacity of steel fiber-reinforced beams, offering a useful tool for design applications.

Although this study provides valuable insights into the shear performance of rubberized and layered concrete beams, it is limited to short-term mechanical performance under monotonic loading. Long-term effects such as durability, fatigue behavior, and environmental exposure were not addressed and should be examined in future research. Furthermore, future studies should explore a broader range of rubber replacement levels, steel fiber contents, and fiber types to better understand their influence on mechanical and shear performance. Another limitation of this study lies in the number of specimens per configuration, which was constrained by practical considerations. While three cylindrical replicates were used for material characterization, only one beam was tested for each configuration. Consequently, the statistical variability in structural performance could not be fully evaluated. Future investigations should therefore increase the number of beam specimens and incorporate statistical analyses to enhance the reliability and generalizability of the findings. The influence of varying the beam aspect ratios, the thickness of layered zones, and the response of deep beams and elements with shear reinforcement (e.g., stirrups) also merits further investigation. Future studies should also consider interface bond testing or embedded sensors to quantify slip and better understand interlayer shear transfer mechanisms. Furthermore, numerical and analytical modeling is also recommended in future work to complement experimental work and to simulate complex phenomena such as nonlinear behavior, stress distribution, crack propagation, and interfacial bond performance in both homogeneous and layered concrete systems. Such models will be essential in optimizing fiber orientation, predicting long-term performance, and guiding the structural implementation of sustainable concrete technologies. It is concluded that the use of waste rubber particles in large quantities in concrete beams did not compromise their shear performance, either when steel fibers were added or in layered concrete.

## Figures and Tables

**Table 1:** Mechanical properties of longitudinal steel reinforcement bars.

Bar Diameter	Elastic Modulus (GPa)	Yield Strength (MPa)	Ultimate Strength (MPa)
8 mm	190	518.3	528.8
12 mm	201.4	550.2	651.2

**Table 2:** Proportions of various concrete mix (kg/m^3^).

Ingredient/Mix ID		P	RU	SF	RUSF
Cement Type-1 (kg/m^3^)		350	350	350	350
Fine aggregates (kg/m^3^)	FAgg (0–1 mm)	560	448	560	448
	FRub (0–1 mm)	0	29.2	0	29.2
	FAgg (1–5 mm)	240	192	240	192
	FRub (1–5 mm)	0	12.5	0	12.5
Coarse aggregate (kg/m^3^)	CAgg (5–10 mm)	330	264	330	264
	CRub (5–10 mm)	0	18	0	18
	CAgg (10–20 mm)	715	572	715	572
	CRub (10–20 mm)	0	39	0	39
W (g/m^3^)		140	140	140	140
HRWRA (L/m^3^)		2.8	3.5	4.4	5.3
RSF (kg/m^3^)		0	0	20	20
ISF (kg/m^3^)		0	0	20	20

**Table 3:** Specifications of the examined beams.

**Beam Designation**	**Beam Geometry (mm)**			**Section Composition**	**Type of Concrete Mixture** **(% from the Beam Thickness) [Thickness in mm]**	
	**L**	**b**	**h**		**Top Layer ^a^**	**Bottom Layer ^b^**
100P	1500	120	180	Homogeneous	P (100%) [180]	
100RU	1500	120	180	Homogeneous	RU (100%) [180]	
100SF	1500	120	180	Homogeneous	SF (100%) [180]	
100RUSF	1500	120	180	Homogeneous	RUSF (100%) [180]	
80RU20P	1500	120	180	Layered	P (20%) [36]	RU (80%) [144]
60RU40P	1500	120	180	Layered	P (40%) [72]	RU (60%) [108]
60RUSF40P	1500	120	180	Layered	P (40%) [72]	RUSF (60%) [108]

^a^ Top layer thickness measured from the top of the beam. ^b^ Bottom layer thickness measured from the bottom of the beam.

**Table 4:** Summary of mechanical properties for the different concrete mixes.

Mix/Property	#	P	RU	SF	RUSF
Compressive strength (MPa)	1	47.5	27.4	46.8	34.1
	2	49.0	29.0	48.3	30.6
	3	48.8	24.5	48.9	31.8
	Ave.	48.4	27.0	48.0	32.2
	STDV.	0.81	2.28	1.08	1.78
Modulus of elasticity (GPa)	1	30.0	21.7	32.5	22.9
	2	32.3	21.2	31.8	21.4
	3	31.0	20.4	31.7	23.6
	Ave.	31.1	21.1	32.0	22.6
	STDV.	1.15	0.66	0.44	1.12
Strain at peak comp. strength	1	2245	2040	3195	2085
	2	2265	1790	2965	2240
	3	2390	1820	2945	2015
	Ave.	2300	1883	3035	2113
	STDV.	78.58	136.50	138.92	115.14
Splitting tensile strength (MPa)	1	5.95	3.64	7.53	6.27
	2	5.47	4.05	6.92	5.60
	3	5.18	4.15	6.56	5.99
	Ave.	5.53	3.95	7.00	5.95
	STDV.	0.39	0.27	0.49	0.34

**Table 5:** Summary of force–displacement and strains at peak load.

Beam ID	at Peak			
	Load	Mid-Span Displacement	Strain at Tension Steel	Strain at Compression Steel
	(kN)	(mm)	Micro-Strain	Micro-Strain
100P	63.4	7.2	2348	297
100RU	44.0	4.1	1936	-
100SF	88.5	17.8	30,241	-
100RUSF	83.7	8.6	5697	-
80RU20P	58.7	7.2	2488	645
60RU40P	63.0	7.3	2014	175
60RUSF40P	68.2	5.1	2682	281

**Table 6:** Predicted vs. experimental shear strength for the beams 100P and 100RU.

Beam No.	V_exp._ (kN)	ACI 318 [50]		Eurocode [51]		Zsutty [52]	
		V_pred._	V_pred._/V_exp._	V_pred._	V_pred._/V_exp._	V_pred._	V_pred._/V_exp._
100P	31.7	20.75	0.65	25.03	0.79	22.33	0.70
100RU	22.0	15.81	0.72	20.60	0.94	18.39	0.84

**Table 7:** Predicted vs. experimental shear strength for the beams 100SF and 100RUSF.

Beam No.	V_exp._ (kN)	Narayanan and Darwish [53]–I *		Narayanan and Darwish [53]–II **	
		V_pred._	V_pred._/V_exp._	V_pred._	V_pred._/V_exp._
100SF	44.3	35.1	0.79	46.1	1.04
100RUSF	41.9	30.8	0.74	41.7	1.00

* The prediction is based on the estimated value of $f_{spfc}$; ** The prediction is based on the experimental value of $f_{spfc}.$

## Data Availability

The original contributions presented in this study are included in the article. Further inquiries can be directed to the corresponding authors.

## Nomenclature

FAgg	Natural fine aggregate
CAgg	Natural coarse aggregate
DTR	Discarded tire rubber
RU	Rubberized concrete
FRub	Fine rubber
CRub	Coarse rubber
ISF	Industrial steel fibers
RSF	Recycled tire steel fibers
RUSF	Rubberized steel fiber reinforced
SF	Steel fiber reinforced
P	Plain
BSG	Bulk specific gravity
LBD	Loose bulk density
PSD	Particle size distribution
WA	Water absorption
W	Mixing water
HRWRA	High-range water-reducing admixture
LVDT	Linear variable differential transducer
SG-B, SG-T	Electrical resistance strain gauges bottom and top
